# Piecewise Sliding-Mode-Enhanced ADRC for Robust Active Disturbance Rejection Control Against Internal and Measurement Noise

**DOI:** 10.3390/s25196109

**Published:** 2025-10-03

**Authors:** Shengze Yang, Junfeng Ma, Dayi Zhao, Chenxiao Li, Liyong Fang

**Affiliations:** 1 School of Aeronautics and Astronautics, University of Electronic Science and Technology of China, Chengdu 611731, China; 202321100314@std.uestc.edu.cn (S.Y.);; 2 Aircraft Swarm Intelligent Sensing and Cooperative Control Key Laboratory of Sichuan Province, Chengdu 611731, China; 3 National Key Laboratory of Adaptive Optics, Chengdu 611731, China; 4 Yangtze Delta Region Institute (Huzhou), University of Electronic Science and Technology of China, Huzhou 313001, China

**Keywords:** robust control, composite control strategy, piecewise SMC, ADRC, EKF

## Abstract

To address the challenges of insufficient response speed and robustness in optical attitude control systems under highly dynamic disturbances and internal uncertainties, a composite control strategy is proposed in this study. By integrating the proposed piecewise sliding control (P-SMC) with the improved active disturbance rejection control (ADRC), this strategy achieves complementary performance, which can not only suppress the disturbance but also converge to a bounded region fast. Under highly dynamic disturbances, the improved extended state observer (ESO) based on the EKF achieves rapid response with amplified state observations, and the Nonlinear State Error Feedback (NLSEF) generates a compensation signal to actively reject disturbances. Simultaneously, the robust sliding mode control (SMC) suppresses the effects of system nonlinearity and uncertainty. To address chattering and overshoot of the conventional SMC, this study proposes a novel P-SMC law which applies distinct reaching functions across different error bands. Furthermore, the key parameters of the composite scheme are globally optimized using the particle swarm optimization (PSO) algorithm to achieve Pareto-optimal trade-offs between tracking accuracy and disturbance rejection robustness. Finally, MATLAB simulation experiments validate the effectiveness of the proposed strategy under diverse representative disturbances. The results demonstrate improved performance in terms of response speed, overshoot, settling time and control input signals smoothness compared to conventional control algorithms (ADRC, C-ADRC, T-SMC-ADRC). The proposed strategy enhances the stability and robustness of optical attitude control system against internal uncertainties of system and sensor measurement noise. It achieves bounded-error steady-state tracking against random multi-source disturbances while preserving high real-time responsiveness and efficiency.

## 1. Introduction

Optical attitude control systems have attracted widespread interest due to their capability for high-precision target tracking and stable imaging across diverse motion platforms, with attitude stability recognized as a critical performance metric [1,2]. In actual control system, practical operating environments frequently impose highly dynamic disturbances, while the internal uncertainties which are caused by assembly misalignments and the external measurement noise of sensor further suppress system robustness [3]. Therefore, this study focuses on developing an optimized composite control strategy for position and attitude regulation under the various representative disturbances, to enhance both disturbance rejection robustness and convergence speed.

Considering the generality of nonlinear, time-varying disturbances, precise modeling and active disturbance rejection of both internal uncertainties and external disturbances have become essential for improving system performance. In industry, muti-source disturbances are often addressed by integrating PID control with robust techniques like fuzzy logic [4,5]. However, these composite methods typically involve intricate design procedures and parameter tuning that rely heavily on empirical expertise, making it difficult to strike an optimal balance between rapid response and robustness.

Active disturbance rejection control (ADRC), as a robust control method specifically designed for nonlinear systems with uncertainties [6], has experienced rapid development owing to its superior disturbance rejection capability and tracking accuracy compared to conventional PID-based methods [7,8]. Although the ADRC is theoretically capable of rejecting a wide range of disturbance types, its inherently nonlinear structure and the need to tune multiple parameters pose significant practical challenges. Gao [9] proposed the linear ADRC (LADRC), which linearizes and parameterizes the observer. By configuring a set of bandwidth parameters, LADRC maintains robust performance against model uncertainties. Due to its concise structure and excellent efficacy, LADRC has seen extensive application in a variety of uncertain systems [10,11,12,13,14,15,16] and has been further extended to the control of fractional-order dynamics [17,18,19], demonstrating its adaptability and flexibility across complex and diverse environments.

Nevertheless, despite its widespread adoption in uncertain systems, ADRC performance remains constrained by two intrinsic limitations. First, the extended state observer (ESO) inevitably incurs non-negligible estimation errors when tracking time-varying unknown disturbances. Second, due to modeling inaccuracies, the gain adjustments of ADRC frequently fail to precisely match the evolving gain requirements of complex dynamic processes [20,21]. As the core of ADRC, ESO treats internal and external disturbances as total disturbances which are estimated by the extended state, and the Nonlinear State Error Feedback (NLSEF) generates a compensation signal to compensate for them in the control law to eliminate their effects. The estimation accuracy of ESO significantly influences system tracking and disturbance rejection performance. Therefore, the introduction of observers such as the Anti Disturbance Extended State Observer (ADESO) [22] and the Model Assisted Extended State Observer (MESO) [23] has significantly enhanced disturbance rejection ability through more precise estimation of uncertainties. Ref. [24] integrates an extended state filter (ESF) into ADRC and proposes an improved ADRC method which improves the stability of closed-loop control systems and reduces the delay between system output and the measurement of sensor input. Adaptive ESO (AESO) [25] employs the recursive parameter tuning mechanism of the EKF to effectively suppress errors arising from model uncertainties and sensor noise. In addition, other AESO variants [26,27,28] have improved the disturbance estimation accuracy. Some studies [29,30] also employ variants of the Kalman filter to improve ESO performance by delivering smoothed state estimates to ADRC, thereby significantly enhancing the system’s robustness against both the measurement noise of sensors and external disturbances. However, these methods typically rely on fixed gains, which limits their capability to dynamically adjust sensitivity to time-varying disturbances and noise. Recent methods have sought to address this limitation through adaptive approaches such as AEKF [31], which enables real-time adjustment of observer parameters for improved tracking of varying uncertainties and noise conditions. Additionally, the cascade ESO (CESO) [32,33] incorporates progressively increasing bandwidth and residual driven iterative estimation, which effectively mitigates the cumulative impact of measurement noise. Ref. [34] proposes a CESO which decomposes the total disturbance into distinct frequency band components, and these components are estimated by different stages of CESO. Specifically, the first stage estimates the total disturbance and provides feedback to the system. Subsequent stages utilize residuals form the preceding stage alongside virtual control signals to perform progressive estimation. The CESO effectively prevents the iterative amplification of high-frequency noise, enhancing the stability and accuracy of the estimation process.

The SMC [35,36] has gained extensive interest owing to the rapid response to the system and robustness against model uncertainties and external disturbances. The classic first-order SMC employs a discontinuous switching function to guarantee that the system state converges to the sliding surface in finite time [37]. Despite its effectiveness, this method frequently results in high frequency chattering and abrupt changes in the control input signals, which degrade system performance. Since the introduction of the fast terminal sliding mode control (FTSMC) [38] and super-twisting sliding mode control (ST-SMC) [39], SMC has realized substantial enhancements in its overall performance. Among them, regarding the singularity issues inherent in traditional terminal SMC, a nonsingular terminal sliding mode (NTSM) surface is introduced [40]. By avoiding denominator singularity, NTSM achieves global approximate convergence within a fixed time, and improves both transient response speed and steady state tracking accuracy. However, a critical challenge in SMC design is balancing convergence speed with system stability. Prioritizing rapid convergence often amplifies overshoot and chattering, whereas an excessively high convergence rate may induce overshoot, and a low rate can cause sluggish system response. To address these limitations, piecewise sliding mode control (P-SMC) strategies have been developed [41,42]. These methods employ high-power functions outside the boundary layer to accelerate convergence, switching to low-power or linear functions within the layer to ensure smooth and stable control. This adaptive method effectively mitigates chattering while maintaining rapid response. Ref. [43] proposes a piecewise SMC strategy based on piecewise power function, while maintaining rapid response characteristics, markedly suppresses chattering and enhances both velocity estimation accuracy and overall system stability. Ref. [44] introduces a piecewise fast muti-power reaching law (PFMPRL), which optimizes convergence speed and chattering suppression by a dual mode switching mechanism. When the system state is far from the sliding surface, PFMPRL employs a dual power reaching law to accelerate convergence; as the state approaches the sliding surface, it switches to a fast power reaching law to reduce chattering and improve control precision.

Although ADRC has advantages in estimating uncertainties and compensating for disturbance, it exhibits a relatively slower system response. To achieve rapid and accurate performance, the SMC is employed to provide fast response and convergence. Therefore, the composite control strategy that combines SMC and ADRC can significantly improve the control accuracy of the system. Ref. [45] proposes a novel composite control strategy based on ADRC and FTSMC, which remarkably reduce the oscillation phenomena and achieve finite-time convergence. Moreover, Ref. [46] proposes a UAV pose control method based on a fusion of ADRC and ST-SMC, achieving strong dynamic and static control performance under random airflow disturbances. Building on these concepts, this study designs a composite control strategy that integrates a piecewise SMC with ADRC, aiming to achieve both rapid response and robust disturbance suppression in complex dynamic environments.

The main contributions of the study are listed as follows.

(1) The P-SMC-ADRC composite control strategy based on ADRC and P-SMC is developed to improve the system robustness and disturbance rejection ability, achieving complementary performance in complex disturbance environments.
(2) A novel piecewise sliding mode controller is proposed, which employs domain-specific reaching laws, ensuring finite-time convergence with minimal overshoot across distinct system deviation conditions.
(3) Improved ESO based on EKF reduces the impact of model uncertainty and sensor measure noise on disturbance estimation, concurrently improving estimation accuracy and response performance.
(4) This study incorporates a particle swarm optimization (PSO) [47] algorithm for automated tuning of the composite strategy’s parameters, to simplify the controller implementation process, enhance the efficiency and convenience of the tuning procedure.

The rest of the paper is organized as follows. Section 2 presents an overview of the proposed composite control framework and provides a detailed exposition of the ESO design optimized by the EKF along with the novel piecewise SMC method. Section 3 proves the stability of the proposed method using Lyapunov theory. Section 4 employs the PSO to globally tune the key parameters in the control law. In Section 5, simulations are conducted to validate the superiority of the proposed strategy through comparative experiments under various disturbance conditions. Section 6 provides a conclusion.

## 2. The Proposed Composite Control Strategy and Component Design

For an optical attitude control system, denote the controlled angle as *θ*, and let the system state vector be $x_{1}=\theta$ and $x_{2}=\dot{\theta }$. The rigid-body angular dynamics can be expressed as:

(1) $$J\ddot{\theta (t)}=\tau_{act}\left(t\right)+\tau_{dist}\left(t\right)-(l_{1}\dot{\theta \left(t\right)}+l_{2}\theta \left(t\right)+\phi \left(\theta ,\dot{\theta }\right))$$

where *J* is the rigid body moment of inertia; *l* is the equivalent damping coefficient; *l*_2_ denotes damping coefficient; $\phi \left(\theta ,\dot{\theta }\right)$ represents residual nonlinear term of the system caused by friction and higher-order terms, leading to uncertainty; $\tau_{act}\left(\cdot \right)$ denotes the actuator’s output torque and $\tau_{dist}\left(\cdot \right)$ denotes the disturbance torque.

If the actuator is represented dynamically with equivalent gain, then it can be obtained as:

(2) $$\tau_{act}\left(t\right)=K_{u}(\theta ,\dot{\theta })u$$

the function (1) can be equivalently converted to:

(3) $$\ddot{\theta }=-\frac{l_{1}}{J}\dot{\theta }-\frac{l_{2}}{J}\theta -\frac{1}{J}\phi \left(\theta ,\dot{\theta }\right)+\frac{K_{u}\left(\theta ,\dot{\theta }\right)}{J}u+\frac{1}{J}\tau_{dist}(t)$$

To facilitate sub sequent derivations and proofs, Equation (3) is transformed into a general second-order form. The second-order nonlinear system can be described as:

(4) $$\left\{\begin{array}{c} \dot{x_{1}}=x_{2}\\ \dot{x_{2}}=f\left(x\right)+g\left(x\right)u+w \end{array}\right.$$

where $x=(x_{1},x_{2})$ denotes the system state vector $\left(\theta ,\dot{\theta }\right)$, $f\left(x\right)=-\frac{l}{J}\dot{\theta }-\frac{w}{J}\theta -\frac{1}{J}\phi \left(\theta ,\dot{\theta }\right)$ represents the uncertain nonlinear dynamics of the system, $g\left(x\right)=\frac{K_{u}\left(\theta ,\dot{\theta }\right)}{J}$ denotes the control gain, $w=\frac{1}{J}\tau_{dist}(t)$ represents the internal disturbance with $w\sim N\left(0,Q\right)$, and u is the control input signals. To ensure the feasibility and robustness of the subsequent controller design, the following standard assumptions are made:

**Assumption 1.**  *There exists a constant F≥0 such that for any system state* $x\in R^{2}$*, the nonlinear term is bounded by* $\left|f(x)\right|\leq F$.

**Assumption 2.**  *There exist positive constant $g_{m}>0$ and* $g_{M}>0$ *such that for any system state* $x\in R^{2}$*, the control gain satisfies* $0<g_{m}<\left|g(x)\right|<g_{M}$.

For the second-order system subject to model uncertainties and external disturbances, ADRC employs an ESO to achieve real-time estimation of both model uncertainties and external disturbance. The Nonlinear State Error Feedback (NLSEF) actively compensates for these estimated perturbations, while the Tracking Differentiator (TD) mitigates transient shock and smooths control signals. Thereby substantially enhancing both tracking accuracy and disturbance rejection performance. Moreover, SMC constructs a sliding surface s=0, and drives the system trajectory onto this surface, maintaining sliding-mode motion to efficiently suppress model uncertainties and external disturbances while guaranteeing rapid convergence even under large initial errors. But the SMC provides rapid convergence from large initial errors and strong robustness to model uncertainty, but its performance typically depends on a priori disturbance bounds and high switching gains tend to induce chattering. Conversely, the ESO-driven NLSEF supplies accurate, real-time estimation of and compensation for the total disturbance, thereby reducing steady-state errors, while its response to large, abrupt uncertainties can be relatively slower.

**Figure 1:**
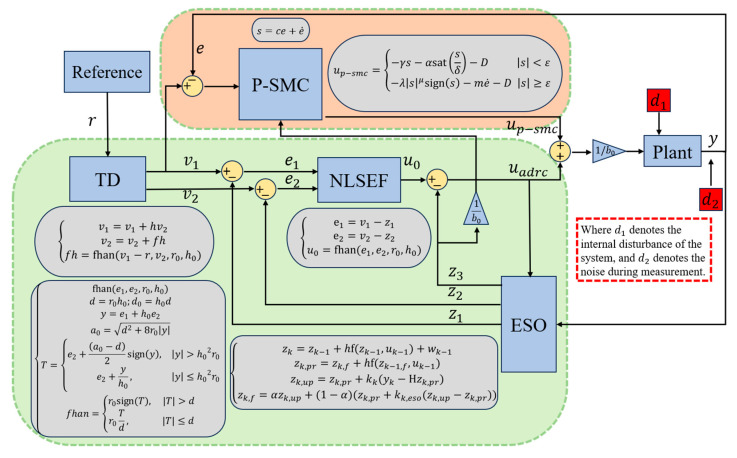
The proposed composite control strategy.

Therefore, a P-SMC-ADRC composite control strategy is proposed as shown in Figure 1. In addition to EKF-ESO based extended state observation with NLSEF for real-time disturbances estimation and compensation, the proposed strategy integrates a piecewise SMC to simultaneously achieve rapid convergence and overshoot suppression, thereby delivering superior overall control performance in complex nonlinear systems against muti-source disturbances. Specifically, the two components play complementary roles rather than acting as independent, simply additive, controllers. Their integration mitigates these drawbacks, by which the disturbance estimated by the EKF-ESO substantially reduces the switching gain required by P-SMC and thus suppresses chattering, while P-SMC guarantees fast, global convergence that secures system performance under large perturbations. However, under the premise of complementarity, the integrated design of P-SMC and NLSEF still needs to independently consider their specific operating conditions.

### 2.1. The ADRC Based on EKF-ESO

**Figure 2:**
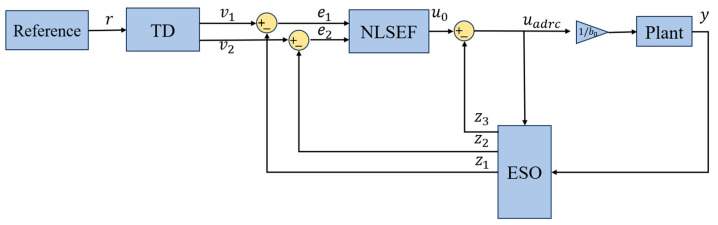
The control architecture for ADRC.

The ADRC estimates disturbances in the I/O signals of plant and generates compensatory control signals before they affect the output, thus minimizing their impact on control performance. A standard ADRC comprises three components: the tracking difference (TD), the extended state observer (ESO) and the nonlinear state error feedback (NLSEF), which is presented in Figure 2.

Among them, TD smooths discontinuous reference signals and accurately estimates signals first derivative to suppress oscillation induced by abrupt setpoint changes; ESO estimates internal dynamics and external disturbances into an extend state; and NLSEF compensates for tracking errors and their high-order derivatives to ensure precise trajectory tracking and system stability, leveraging the ESO estimates. To further improve the performance of the observer, this study introduces an EKF-ESO, which employs the data prediction optimization capability of EKF to refine both state and disturbance estimations in complex environments, by optimizing the observer’s estimation process rather than directly processing the raw sensor measurements.

#### 2.1.1. The Introduction of TD

The TD performs smoothing processing on the original reference signal r(t), while synchronously generating the first order derivative estimation of this reference signal, resulting in a smooth reference signal $v_{1}(t)$ and its approximate derivative $v_{2}(t)$. The specific implementation form of this Tracking Differentiator (TD) can be described as:

(5) $$\left\{\begin{array}{c} v_{1}=v_{1}+hv_{2}\\ v_{2}=v_{2}+fh\\ fh=\mathrm{fhan}(v_{1}-r,v_{2},r_{0},h_{0}) \end{array}\right.$$

where r(t) is the original reference signal, $r_{0}$ denotes the fast tracking factor and $h_{0}$ denotes the filtering factor, which significantly influence the tracking speed of the TD towards the original reference signal r(t), and fhan(·) [9] is given by:

(6) $$\left\{\begin{array}{c} d=r_{0}h_{0};\;d_{0}=h_{0}d\\ y=e_{1}+h_{0}e_{2}\\ a_{0}=\sqrt{d^{2}+8r_{0}\left|y\right|}\\ T=\left\{\begin{array}{cc} e_{2}+\frac{\left(a_{0}-d\right)}{2}sign\left(y\right), & \left|y\right|>{h_{0}}^{2}r_{0}\\ e_{2}+\frac{y}{h_{0}}, & \left|y\right|\leq {h_{0}}^{2}r_{0} \end{array}\right.\\ \mathrm{fhan}=\left\{\begin{array}{ll} r_{0}\mathrm{sign}(T), & |T|>d\\ r_{0}\frac{T}{d}, & |T|\leq d \end{array}\right. \end{array}\right.$$

The Tracking Differentiator (TD) enhances control performance by smoothing the reference signal and providing its derivative, thereby reducing sensitivity to noise and setpoint changes, and improving both convergence speed and robustness.

#### 2.1.2. The Design of ESO

For ADRC, the second-order system (4) can be equivalently converted to the following form

(7) $$\left\{\begin{array}{c} \ddot{x_{1}}=f(x)+g(x)u+w\\ y=Hx+v \end{array}\right.$$

where y denotes the measurement output, and *v* is the measurement noise, with $v\sim N\left(0,R\right),H=[1,0]$.

The conventional ESO is a specialized state observer designed to estimate not only the system states but also an extended state that represents the total disturbance d. This total disturbance is defined as d=w+v+f(x), which aggregates the collective effect of various uncertainties from different channels, including external disturbances w, sensor measurement noise v, and internal system nonlinearities and uncertainties f(x). Although these components enter the system through distinct pathways, the ESO treats their impact on the controlled dynamics as a single, unified entity to be estimated. The ESO [9] can be described as

(8) $$\left\{\begin{array}{c} \dot{z_{1}}=z_{2}-\beta_{01}(z_{1}-y)\\ \dot{z_{2}}=z_{3}-\beta_{02}(z_{1}-y)+bu\\ \dot{z_{3}}=-\beta_{03}(z_{1}-y) \end{array}\right.$$

where $z=(z_{1},z_{2},z_{3})$ denotes the estimates of the extended states $x_{1},x_{2},x_{3}$, which represent the system output, its derivative, and the total disturbance comprising both internal uncertainties and external disturbances, respectively; the gain matrix is chosen as $L={\left[\beta_{01},\beta_{02},\beta_{03}\right]}^{T}=[3\omega_{0}{,3{\omega_{0}}^{2},{\omega_{0}}^{3}]}^{T}$.

To facilitate digital implementation, the continuous observer is discretized by a forward-difference scheme with sampling period h [9]

(9) $$\left\{\begin{array}{c} z_{1,k}=z_{1,k-1}+h\left[z_{2,k-1}-\beta_{01}e_{1}\right]\\ z_{2,k}=z_{2,k-1}+h\left[z_{3,k-1}-\beta_{02}fe+bu\right]\\ z_{3,k}=z_{3,k-1}-h\beta_{03}fe1 \end{array}\right.$$

where $e_{1}$, fe and fe1 are defined in Equation (7),

(10) $$\left\{\begin{array}{c} e_{1}=x_{1}-y\\ fe=fal\left(e_{1},0.5,\delta \right)=\left\{\begin{array}{cc} \frac{e_{1}}{\delta^{0.5}} & \left|e_{1}\right|\leq \delta \\ \mathrm{sign}\left(e_{1}\right){\left|e_{1}\right|}^{0.5} & \left|e_{1}\right|>\delta \end{array}\right.\\ fe1=fal\left(e_{1},0.25,\delta \right)=\left\{\begin{array}{cc} \frac{e_{1}}{\delta^{0.25}} & \left|e_{1}\right|\leq \delta \\ \mathrm{sign}\left(e_{1}\right){\left|e_{1}\right|}^{0.25} & \left|e_{1}\right|>\delta \end{array}\right. \end{array}\right.$$

#### 2.1.3. Introduction of EKF

To enhance the robustness and accuracy of the ESO estimation, this study incorporates an EKF to optimize the ESO outputs. Equation (9) can be rewritten in the following form.

(11) $$\left\{\begin{array}{c} z_{k}=Az_{k-1}+Bu_{k-1}\\ x_{k}=z_{k}+{w_{e\_k}}^{\prime } \end{array}\right.$$

where the true system state vector $x_{k}\in R^{3\times 1}$, the observer’s estimated state vector $z_{k}\in R^{3\times 1}$ $A=\left[\begin{array}{ccc} 1-h\beta_{01} & h & 0\\ -h\beta_{02}k_{1} & 1 & h\\ -h\beta_{03}k_{2} & 0 & 1 \end{array}\right],$ $B=\left[\begin{array}{cc} 0 & h\beta_{01}\\ b & h\beta_{02}k_{1}\\ 0 & h\beta_{03}k_{2} \end{array}\right]$, $w_{e\_k}’$ represents the error between the observed value and the true state, and $k_{1}$ and $k_{2}$ denote the approximated linear gains of the nonlinear functions fe and fe1, respectively.

For the classical Kalman filter,

(12) $$\left\{\begin{array}{c} {\hat{z}}_{k,up}={\hat{z}}_{k,pr}+k_{k}\left(y_{k}-H^{\prime }{\hat{z}}_{k,pr}\right)\\ e_{k}=x_{k}-{\hat{z}}_{k,up}\\ e_{k,pr}=x_{k}-{\hat{z}}_{k,pr} \end{array}\right.$$

where $H^{\prime }=\left[\begin{array}{ccc} 1 & 0 & 0 \end{array}\right]$. $e_{k}$ is estimation error, with $e_{k}\sim N\left(0,p\right)$. $p=E\left[e_{k}e_{k}^{T}\right]$, which denotes the error covariance. By appropriately designing the gain $k_{k}$, the variance p of the estimation error $e_{k}$ is minimized, thereby ensuring that the EKF-ESO estimated state ${\hat{z}}_{k}$ closely approximates the system true state $x_{k}$. To find the gain $k_{k}$, the extremum of $tr\left(p\right)$ is calculated, which $\frac{dtr\left(p\right)}{dk_{k}}=0$. Eventually, the gain $k_{k}$ is obtained as

(13) $$k_{k}=p_{k,pr}H^{\prime T}{\left(H^{\prime }p_{k,pr}H^{\prime T}+R\right)}^{-1}$$

To account for the influence of external disturbances, the error covariance matrix is augmented as

(14) $$p_{k,pr}=Ap_{k-1}A^{T}+Q$$

where R is the measurement noise covariance matrix, with $R\in R^{1\times 1}$, Q denotes process noise covariance matrix, with $Q\in R^{3\times 3}$. It is worth noting that the matrix Q should be symmetric positive definite.

Therefore, the main steps are summarized as

(15) $$\left\{\begin{array}{c} {\hat{z}}_{k,pr}=A{\hat{z}}_{k-1}+Bu_{k-1}\\ p_{k,pr}=Ap_{k-1}A^{T}+Q\\ k_{k}=p_{k,pr}H^{\prime T}{\left(H^{\prime }p_{k,pr}H^{\prime T}+R\right)}^{-1}\\ {\hat{z}}_{k,up}={\hat{z}}_{k,pr}+k_{k}\left(z_{k}-H^{\prime }{\hat{z}}_{k,pr}\right)\\ p_{k}=(I-k_{k}H^{\prime })p_{k,pr} \end{array}\right.$$

To enhance the accuracy of the ESO, a scale factor κ is introduced to current the ESO output using the EKF covariance,

(16) $$\left\{\begin{array}{c} z_{eso}=z_{pr}+k_{eso}(z_{up}-z_{pr})\\ k_{eso}=\kappa \times diag(\frac{1}{diag(p_{pr})+1\times 10^{-6}}) \end{array}\right.$$

The covariance matrix of the EKF reflects the uncertainty of the estimates, providing a more reliable basis for the correction process. Afterwards, the corrected ESO estimates are weighted and fused with the EKF updated values,

(17) $$z_{f}=\alpha z_{up}+(1-\alpha )z_{eso}$$

In conclusion, the complete algorithm EKF-ESO is outlined below,

(18) $$\left\{\begin{array}{c} z_{k}=z_{k-1}+hf(z_{k-1},u_{k-1})+w_{k-1}\\ z_{k,pr}=z_{k,f}+hf(z_{k-1,f},u_{k-1})\\ z_{k,up}=z_{k,pr}+k_{k}(y_{k}-H^{\prime }z_{k,pr})\\ z_{k,f}=\alpha z_{k,up}+(1-\alpha )(z_{k,pr}+k_{k,eso}(z_{k,up}-z_{k,pr})) \end{array}\right.$$

#### 2.1.4. The Design of NLSEF

Based on the EKF-ESO estimation output, the feedback control law is formulated using the NLSEF as

(19) $$u_{adrc}=-\frac{1}{b_{0}}\left(u_{0}-D\right)$$

where $u_{0}=fhan(e_{1},e_{2},r_{0},h_{0})$ denotes the NLSEF output with $e_{1}$ and $e_{2}$ representing the tracking error and its derivative, $r_{0}$ and $h_{0}$ being tunable parameters that adjust the convergence rate and damping characteristics; $D=z_{3}=\hat{f}\left(x\right)+\hat{d}\left(t\right)$ is the estimated total disturbance; and the normalization factor $b_{0}$ ensures that the control input signals u accurately compensates for the system’s nonlinear dynamics.

### 2.2. The Piecewise SMC

**Figure 3:**
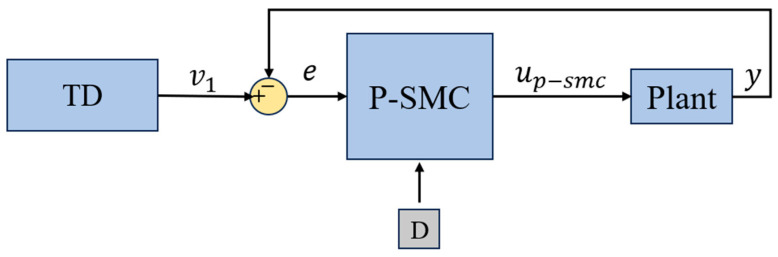
The control architecture for piecewise SMC.

SMC guides system states onto a predefined sliding surface and maintains motion along it by applying high-gain switching feedback on either side of the surface, thereby effectively rejecting model uncertainties and external disturbances. However, frequent high-gain switching often induces chattering and overshoot. To address these drawbacks, this study proposes a novel error-region-based piecewise SMC (P-SMC) law for second-order nonlinear systems, as shown in Figure 3.

By employing the smoothed reference signal $v_{1}(t)$ generated by the Tracking Differentiator (TD) as reference signal, as expressed in Equation (5), the noise in the reference signals is significantly attenuated. For the piecewise SMC, when the tracking error exceeds a prescribed threshold, a high-gain switching mechanism is activated to guarantee rapid convergence; once the error enters a small region, the switching gain is reduced and a continuous approximation function is employed to smooth the control input signals, thus suppressing chattering and limiting overshoot. The piecewise control method balances rapid response with minimal overshoot, significantly enhancing the robustness of SMC under complex dynamic conditions.

#### The Design of P-SMC

The sliding surface is defined as follows,

(20) $$s=ce+\dot{e}$$

where $e=x_{1}-v_{1}$ denote the tracking error, r is the reference signal, c is the sliding surface gain, which ensures that the system has the desired dynamic characteristics when s=0.

To satisfy the requirements of dynamic response, a power rate reaching law is adopted for the sliding mode control,

(21) $$\dot{s}=-\lambda {\left|s\right|}^{\mu }sign(s)$$

where λ>0 is the reaching law gain, 0<μ<1 is the index parameters.

So, the sliding mode control law is given by

(22) $$u_{smc1}=-\lambda {\left|s\right|}^{\mu }sign(s)$$

Although classical high-gain switching laws achieve rapid convergence once the system state reaches the sliding surface, the associated frequent high-gain actions often induce chattering and excessive overshoot, substantially degrading closed-loop performance. To address these issues, this study introduces an improved piecewise sliding mode control law,

- Large error region: when $\left|s\right|\geq \varepsilon$, a power rate reaching law combined with linear feedback is applied to swiftly drive the state onto the sliding surface:
  (23) $$u_{L-smc}=-\lambda {\left|s\right|}^{\mu }sign(s)-m\dot{e}$$
- Boundary layer region: when $\left|s\right|<\varepsilon$, a linear sliding mode form is used with a continuous saturation function replacing the signum to smooth the control input signals and suppress chattering:
  (24) $$u_{B-smc}=-\gamma s-\alpha sat(s)$$
  where λ and m denote the power law and linear feedback gains; μ is the exponent parameter; γ is the boundary layer gain; α denotes the gain for suppressing chattering; sat(·) is the saturation function, defined as
  (25) $$sat(s)=\left\{\begin{array}{c} \mathrm{sign}(s)\left|s\right|\geq \delta \\ \frac{s}{\delta }\;\;\;\;\;\;\;\;\;\;\;\left|s\right|<\delta \end{array}\right.$$

Here, 0<δ<ε in the saturation function is a positive parameter used to control the smoothness of the sliding mode control law.

Considering the effects of internal uncertainties within the system and external disturbances, this study introduces the perturbation D estimated by EKF-ESO. Design the total piecewise sliding mode control u as follows,

(26) $$u_{P-SMC}=\left\{\begin{array}{c} -\gamma s-\alpha \mathrm{sat}(s)-D\\ -\lambda {\left|s\right|}^{\mu }\mathrm{sign}(s)-m\dot{e}-D \end{array}\right.\begin{array}{c} \left|s\right|<\varepsilon \\ \left|s\right|\geq \varepsilon \end{array}$$

This piecewise control mechanism balances rapid response with input signals smoothness, markedly enhancing robustness and stability under complex nonlinear dynamic and stochastic disturbances.

### 2.3. The Composite Control Law Design

By integrating ADRC with P-SMC, the composite control law is formulated as follows,

(27) $${u=u_{adrc}+u}_{S-SMC}=\left\{\begin{array}{c} -\frac{1}{b_{0}}(u_{0}+\gamma s+\alpha \mathrm{sat}(\frac{s}{\delta })+D)\\ -\frac{1}{b_{0}}(u_{0}+\lambda {\left|s\right|}^{\mu }\mathrm{sign}(s)+m\dot{e}+D) \end{array}\right.\begin{array}{c} \left|s\right|<\varepsilon \\ \left|s\right|\geq \varepsilon \end{array}$$

where a single total-disturbance estimation *D* compensates both sub-controllers, thereby reducing observer workload and enhancing overall control performance.

## 3. The Stability Analysis

- **ADRC stability analysis:**

Consequently, the ADRC framework effectively attenuates significant portions of unknown dynamic disturbances. Since EKF-ESO solely enhances estimation accuracy without modifying the controller structure, the closed-loop stability follows directly from the standard ADRC proof methodology [48].

- **P-SMC stability analysis:**

Consider the Lyapunov function as follows.

(28) $$V=\frac{1}{2}s^{2}$$

Based on system Equations (4) and (20), its time derivative is formulated as below,

(29) $$\dot{V}=s\dot{s}=s(c\dot{e}+\ddot{e})=sc\dot{e}+s\left(f\left(x\right)+g\left(x\right)u\right)-s\ddot{v_{1}}$$

where $s=ce+\dot{e}$; $e=x_{1}-v_{1}$.

When $\left|s\right|\geq \varepsilon$, considering Equation (26) and total disturbance, the $\dot{V}$ is modified as follows,

(30) $$\dot{V}=s\dot{s}=cs\dot{e}+sf\left(x\right)-\frac{g(x)}{b_{0}}\lambda {\left|s\right|}^{\mu +1}-\frac{g(x)}{b_{0}}ms\dot{e}-\frac{g(x)}{b_{0}}sD-s\ddot{v_{1}}+sd(t)$$

**Remark 1.**  g(x) *denotes the gain of control input signals, the normalization* *b*_0_ *approximates the unknown system control gain* g(x)*. The reference signal* *v*_1_*, smoothed by the tracking differentiable (TD) component of the ADRC, exhibits no abrupt changes. It is assumed to be twice differentiable with a bounded second derivative, which there exists a positive constant* $v_{0}>0$*, such that* $\left|\ddot{v_{1}}\right|\leq v_{0}$.

Equation (30) is modified as follows,

(31) $$\dot{V}=(c-m)s\dot{e}+s(f\left(x\right)+d(t)-D)-\lambda {\left|s\right|}^{\mu +1}-s\ddot{v_{1}}$$

Obviously, when the parameters are set reasonably, it can eliminate the interference terms, and the following can be obtained

(32) $$\dot{V}<-\lambda {\left|s\right|}^{\mu +1}+\left|s\right|(v_{0}+∆)$$

Here, $∆\;=f\left(x\right)+d(t)-D$ represents the residual uncompensated perturbation arising from the estimation error of the total perturbation by the EKF-ESO. This residual perturbation is characterized as a bounded uncertainty satisfying $\left|∆\right|<\varrho$. For the larger error region $\left|s\right|\geq \varepsilon$, it follows that if the parameter λ satisfies

(33) $$\lambda >\frac{v_{0}+\varrho }{\varepsilon^{\mu }}$$

Then $\dot{V}<0$, ensuring that the system state converges in finite time to the boundary layer $\left|s\right|\leq \varepsilon$.

When $\delta \leq \left|s\right|\leq \varepsilon$, based on Equation (26), the $\dot{V}$ is modified as follows,

(34) $$\dot{V}=cs\dot{e}+sf\left(x\right)-\frac{g(x)}{b_{0}}\gamma s^{2}-s\frac{g(x)}{b_{0}}\alpha sat(s)-\frac{g(x)}{b_{0}}sD-s\ddot{v_{1}}+sd(t)$$

Because a continuous saturation function is employed, for the region $\delta \leq \left|s\right|\leq \varepsilon$, the control law reduces to the large error form. At this stage, the power rate reaching law has already driven the system state close to the reference, so it is reasonable to assume the tracking error satisfies $\left|e\right|<E$, the $\dot{V}$ can be written as

(35) $$\dot{V}=-\left(\gamma -c\right)s^{2}-\alpha \left|s\right|-c^{2}se-s\ddot{v_{1}}+s∆$$

Therefore, if the parameters are satisfied

(36) $$\gamma >c,\;\alpha >c^{2}E+v_{0}+\varrho$$

Then $\dot{V}\leq 0$, ensuring that the state converges further into the boundary layer $\left|s\right|\leq \delta$.

Within the boundary layer $\left|s\right|<\delta$, the saturation degenerates to a linear mapping $sat(s)=\frac{s}{\delta }$, and the $\dot{V}$ simplifies to

(37) $$\dot{V}=-\gamma s^{2}-\alpha \frac{s}{\delta }+cs(s-ce)-s\ddot{v_{1}}+s∆$$

At the same time, the system state further converges to a smaller region. Denoting the error bound in this region by $\left|e\right|<E_{1}$,

(38) $$\dot{V}\leq [(c-\gamma )\delta +\beta -\frac{\alpha }{\delta }\frac{s}{\left|s\right|}]\left|s\right|$$

where $\beta =c^{2}E_{1}+v_{0}+\varrho >0$.

**Remark 2.**  *Recognizing that within the boundary layer* $\left|s\right|$ *remains sufficiently small, the term of* $-\alpha \frac{s}{\delta }$ *is designated as the dominant component to ensure linear sliding mode action and smooth control input signals.*

Accordingly, α is chosen to satisfy

(39) $$\alpha >\delta (\left|c-\gamma \right|\delta +\beta )+\eta$$

where η>0 is an arbitrarily small constant, which can be achieved by adjusting α.

Under this condition, the $\dot{V}$ is bounded by

(40) $$\dot{V}\leq -\left[\frac{\alpha }{\delta }-\left|c-\gamma \right|\delta -\beta \right]\left|s\right|\leq -\eta \left|s\right|$$

Since V(s) is positive definite and radially unbounded, for $\left|s\right|\leq \delta$, it follows that

(41) $$\dot{V}\leq -\eta \left|s\right|\leq -\eta \sqrt{2}V^{1/2}$$

By integrating both sides of the inequality presented in Equation (41) with respect to time, it is possible to derive a finite time at which the system tends to a stable value. This time is given by

(42) $$t_{s}=\frac{2V^{1/2}(0)}{\eta \sqrt{2}}$$

For $t\geq t_{s}$, it follows that $V^{1/2}(t)=0$, which directly implies $s(t_{s})=0$.

More generally, the system trajectory decreased monotonically for $\left|s\right|>0$ until entering the ultimate bound set

(43) $$\Omega =\{s|\left|s\right|\leq \omega \},\omega =\frac{\delta }{\alpha }(\left|c-\gamma \right|\delta +\beta )$$

Here the parameters α, γ and the boundary layer thickness δ are adjustable. Through judicious selection of these parameters, the value of ω can be rendered arbitrarily small. This adjustment ensures that the closed loop system satisfied the property of uniform ultimate boundedness.

**Remark 3.**  *Define the boundary threshold as* $\omega =\frac{\delta }{\alpha }(\left|c-\gamma \right|\delta +c^{2}E_{1}+v_{0})$*. By appropriately decreasing δ and increasing α, ω becomes proportional to* $\frac{1}{\alpha }$ *and approaches 0. Since* $E_{1}$ *is typically a small quantity, ensuring* $\alpha >c^{2}E+v_{0}$ *guarantees that* $\left|s\right|$ *converges to an acceptable small interval, further suppressing chattering near the sliding surface.*

When the system state reaches the sliding surface s=0, the relation $s=ce+\dot{e}=0$ implies

(44) $$\dot{e}=-ce$$

The closed loop dynamics reduce to a first-order exponentially convergent subsystem. Given a bounded initial error, this subsystem converges to 0 in finite time and remains on the sliding surface thereafter. In conclusion, by combining the finite time reaching property with exponential stability on the sliding surface, the proposed closed-loop system guarantees uniform ultimate boundedness and exhibits exponential convergence along the sliding manifold.

- **The composite control stability analysis:**

**Remark 4.**  *The ADRC stage effectively eliminates the unknown dynamics of system and disturbances, leaving only the estimation errors* $\tilde{f}=f-\hat{f}$ *and* $\tilde{d}=d-\hat{d}$*, which satisfy* $\left|\tilde{f}\right|\leq \epsilon_{f},\;\left|\tilde{d}\right|\leq \epsilon_{d}$.

When $\left|s\right|\geq \varepsilon$, substituting the composite control law $u=-\frac{1}{b_{0}}(u_{0}+\lambda {\left|s\right|}^{\mu }sign(s)+m\dot{e}+D)$ and the system dynamics (function (1)) into the derivative of the sliding surface s. Additionally, considering Remark 1, function (31) and Reference [48], it can be concluded that after the disturbance is compensated by the NLSEF controller, the remaining uncompensated disturbance is constrained within a specific range. Through parameter tuning, the time derivative of the sliding surface $\dot{s}$ can be rewritten as:

(45) $$\dot{s}=-\lambda {\left|s\right|}^{\mu }sign\left(s\right)+{∆}_{1}-\ddot{v_{1}}$$

where ${∆}_{1}=f\left(x\right)+d\left(t\right)-u_{0}-D$, representing the residual uncompensated disturbance after NLSEF-based compensation, and ${∆}_{1}$ satisfied $\left|{∆}_{1}\right|\leq \epsilon_{f}+\epsilon_{d}<\varrho$.

On the basis of Assumption 1, Remark 1 and Remark 4, the time derivative of the Lyapunov function $\dot{V}$ is derived as:

(46) $$\dot{V}=-\lambda {\left|s\right|}^{\mu +1}-s\ddot{v_{1}}+s{∆}_{1}$$

Consequently, the $\dot{V}$ is bounded by

(47) $$\dot{V}=s\dot{s}\leq -\lambda {\left|s\right|}^{\mu +1}+\left|s\right|v_{0}+\left|s\right|(\epsilon_{f}+\epsilon_{d})$$

Thus, $\lambda {\left|s\right|}^{\mu }sign(s)$ acting as the dominant term, ensures rapid system convergence and counteracts the residual uncertainty ${∆}_{1}$ in the large error region.

When $\left|s\right|<\varepsilon$, substituting the composite control law $u=-\frac{1}{b_{0}}(u_{0}+\gamma s+\alpha sat(\frac{s}{\delta })+D)$, the system dynamics, and function (34) into the derivative of the Lyapunov function. By accounting for Remark 2, the $\dot{V}$ is modified as follows,

(48) $$\dot{V}=-\gamma s^{2}-s\alpha sat(\frac{s}{\delta })+s{∆}_{1}$$

For the term $s\alpha sat(\frac{s}{\delta })$, the following inequality is satisfied for any $\left|s\right|<\varepsilon$:

(49) $$s\alpha sat(\frac{s}{\delta })\geq \frac{{\left|s\right|}^{2}}{max(\left|s\right|,\delta )}\geq \frac{{\left|s\right|}^{2}}{\varepsilon +\delta }$$

For the residual term $s{∆}_{1}$, Young’s inequality is employed. For any constant ι>0, the following relation holds:

(50) $$\left|s{∆}_{1}\right|\leq \frac{\iota }{2}s^{2}+\frac{1}{2\iota }{{∆}_{1}}^{2}$$

As a result, the $\dot{V}$ is bounded by

(51) $$\dot{V}=s\dot{s}\leq -\left(\gamma +\frac{\alpha }{\varepsilon +\delta }-\frac{\iota }{2}\right)s^{2}+\frac{1}{2\iota }{{∆}_{1}}^{2}$$

Let $a=\gamma +\frac{\alpha }{\varepsilon +\delta }-\frac{\iota }{2}$ and $b=\frac{1}{2\varrho_{1}}{∆}^{2}$. To satisfy $\dot{V}\leq 0$, the condition a>0 must be met, which implies ι should satisfy $\iota <2(\gamma +\frac{\alpha }{\varepsilon +\delta })$; therefore, by selecting appropriate values for parameters γ and α, the constraint $\dot{V}\leq 0$ can be fulfilled.

Under this premise, the above inequality for $\dot{V}$ is equivalent to:

(52) $$\dot{V}\leq -aV+b$$

By solving the corresponding homogeneous differential equation to obtain the exponential decay term, the following result is obtained:

(53) $$V\left(t\right)\leq V\left(0\right)e^{-2at}+\frac{b}{2a}(1-e^{-2at})$$

Thus, as t→∞, the following conclusions hold:

(54) $$\underset{t\to \infty }{\lim}\sup\;V(t)\leq \frac{b}{2a}$$

(55) $$\underset{t\to \infty }{\lim}\sup\left|s(t)\right|\leq \frac{\epsilon_{f}+\epsilon_{d}}{\sqrt{2\iota a}}$$

It can therefore be concluded that s(t) is uniformly ultimately bounded for any bounded initial value.

**Remark 5.**  *The P-SMC stage further refines performance through two mechanisms:*

- *Boundary layer region: linear feedback* −γs *and the saturation term* $-\alpha sat(\frac{s}{\delta })$ *dominate, smoothing the control input signals to suppress chattering and limit overshoot;*
- *Large error region: the power rate reaching term* $\lambda {\left|s\right|}^{\mu }\mathrm{sign}(s)$ *ensures rapid convergence and counters residual uncertainties* $\epsilon_{f}$ *and disturbances* $\epsilon_{d}$*, while the damping term* $-m\dot{e}$ *enhance stability.*

Notably, for μ<1, ${\left|s\right|}^{\mu }$ amplifies control effort when the error is large and attenuates it when the error is small, mitigating overshoot; the sat(·) function further reduces chattering, yielding a smoother control action.

## 4. Parameter Tuning Based on PSO

The parameter tuning follows a sequential approach. The EKF-ESO and NLSEF parameters are first optimized to minimize the disturbance estimation error. Following this, the P-SMC parameters, particularly those of the reaching law and boundary layer, are tuned to ensure the switching term acts predominantly on the residual estimation error, thus achieving robust convergence without aggravating chattering or overshoot. However, this parameter tuning is too complicated and involves many parameters. To address the tuning complexity arising from the numerous interdependent parameters in the proposed composite control strategy, the particle swarm optimization (PSO) algorithm [47] is applied for global search and rapid convergence. In a d-dimensional parameter space with swarm size n, each particle i is characterized by a position vector $X_{i}=(x_{i1},x_{i2},\ldots x_{id})$ and a velocity vector $V_{i}=(v_{i1},v_{i2},\ldots v_{id})$, representing its candidate solution and search direction, respectively. At the t-th iteration, particle updates follow

(56) $$V_{id}(t+1)=wV_{id}(t)+c_{1}r_{1}(P_{id}(t)-X_{id}(t))+c_{2}r_{2}(P_{gd}(t)-X_{id}(t))$$

(57) $$X_{id}(t+1)=X_{id}(t)+V_{id}(t+1)$$

where w is the inertia weight, $c_{1}$ and $c_{2}$ are cognitive and social coefficients, $r_{1}$ and $r_{2}$ are independent uniform random variables, and $P_{id}(t)$ and $P_{gd}(t)$ denote the particle’s personal best and the swarm’s global best positions, respectively. If the new position $X_{i}(t+1)$ yields a fitness $J(X_{i}(t+1))$ superior to the particle’s previous best fitness $J_{best}$, the personal best $P_{i}$ is updated accordingly, otherwise, it remains unchanged.

The control performance is quantified by the integral of absolute error (IAE)

(58) $$J=\int_{0}^{t}\left|e(t)\right|dt$$

**Figure 4:**
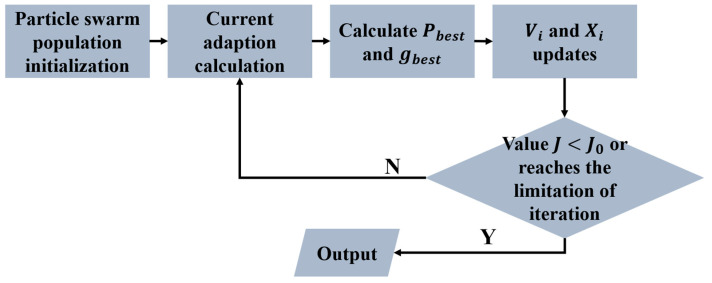
The PSO flowchart for parameters tuning.

The parameter tuning procedure based on PSO, illustrated in Figure 4, iteratively refines the swarm until convergence criteria are met, yielding the optimal set of control parameters.

## 5. Results

To validate the effectiveness of the proposed composite control strategy, simulations are implemented applying MATLAB/SIMULINK 2019b on a complex second-order nonlinear system. Firstly, PSO was employed to tune the controller parameters by minimizing the IAE. Subsequently, under various types and intensities of disturbances, the proposed P-SMC-ADRC composite control strategy was benchmarked against classical control algorithms through comparative simulations.

### 5.1. Experimental Design and Parameters Tuning

Here, a second-order nonlinear system is considered as given below:

(59) $$\left\{\begin{array}{c} \dot{x_{1}}=x_{2}\\ \dot{x_{2}}=20.5\sin(x_{1}+x_{2})-3.41x_{2}+16.2u\\ y=x_{1}+n \end{array}\right.$$

where n denotes the noise of measurement.

The test reference trajectories are defined as the step signal r(t)=35° and the sampling time h was set to 1 ms. Multiple simulation scenarios are designed to evaluate the robustness of various control strategies on a representative second-order nonlinear system. To emulate both random and abrupt disturbances, the following disturbance models are used:

(1) **Gaussian (white noise) noise:** Independent Gaussian noise vectors are superimposed on both the system input and output channels, simulating dynamics process disturbances and sensor measurements noise, respectively, given by Equation (60):
  (60) $$w(t)\sim N(0,\sigma^{2})$$
  where $\sigma^{2}=\frac{N_{0}}{h}$; h is the sample time; $N_{0}$ is the power spectral density and the value of $N_{0}$ is set to 0, 0.1, 1 in this section.
(2) **Sinusoidal (periodic) disturbance:** A sinusoidal disturbance is added to study performance under periodic excitations. The disturbance has an amplitude 10 and frequency 1 Hz. The sinusoidal signal is applied either at the plant input or at the measurement port as indicated in each scenario.
(3) **Pulse (periodic) disturbance:** Pulse disturbances with an amplitude of 10 and a period of 1 s are introduced to emulate transient shocks.
(4) **Random (band-limited) disturbance:** A pseudo-random noise with a mean of 0 and a variance of 20 is used to simulate colored random noise.
(5) **Sudden disturbance:** a sudden disturbance $r_{d}\left(t\right)=-35$ is introduced to assess the response to abrupt disturbance.

Notably, the design and testing of the controller are based on a normalized dimensionless plant dynamic model. This method ensures the generality of the results. To interpret the simulation outcomes in a practical context, the system states are scaled to represent the optical system’s line-of-sight angular position (°). Within the normalized simulation environment, both the control input *u* and the disturbances injected into the input channel are dimensionless. For hardware implementation, the controller’s dimensionless output *u* is mapped to a physical torque command (in N∙m) through a predetermined scaling factor.

Six control methods were compared in a closed-loop:

- Classic ADRC;
- Cascaded ADRC(C-ADRC);
- The Nonsingular terminal SMC(T-SMC) [49];
- The proposed piecewise SMC(P-SMC);
- The composite control based on the Nonsingular terminal SMC(T-SMC-ADRC);
- The proposed P-SMC-ADRC.

In the baseline T-SMC, the sliding variable is constructed on the Nonsingular terminal form:

(61) $$s=x+\rho {\dot{x}}^{\eta }$$

where ρ>0, $1<\eta =\frac{p}{q}<2$.

The reaching law is chosen as in Equation (21), which guarantees that the system trajectory is driven onto this manifold within finite time.

To compensate for disturbances and normalize by the control gain $b_{0}$, the T-SMC law is specified as:

(62) $$u_{T-SMC}=-\frac{1}{b_{0}}(Dsat(s)+\frac{b_{0}}{\eta }({x_{2}}^{2-\eta }+\rho {\left|s\right|}^{\sigma }sat(s){x_{2}}^{1-\eta }))$$

where sat(·) denotes the continuous saturation function to mitigate chattering, σ, ρ and η are design parameters tuned to balance convergence speed and robustness.

**Figure 5:**
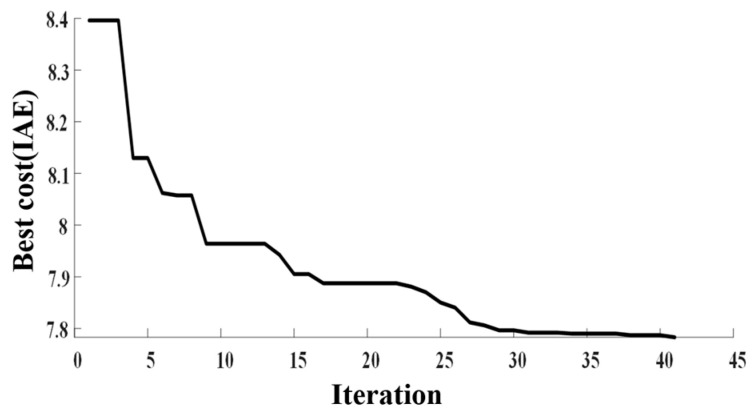
Fitness convergence.

As shown in Figure 5, the fitness curve exhibits a marked slowdown in descent around the 28th iteration, converging at IAE = 7.8.

By applying PSO for parameter tuning of the proposed composite strategy, the optimal parameters of the controller can be obtained, as shown in Table 1.

The optimal parameters of the T-SMC are shown in Table 2.

The optimal parameters of the ADRC and C-ADRC are shown in Table 3.

### 5.2. Comparison of Sliding Mode Control Methods

**Figure 6:**
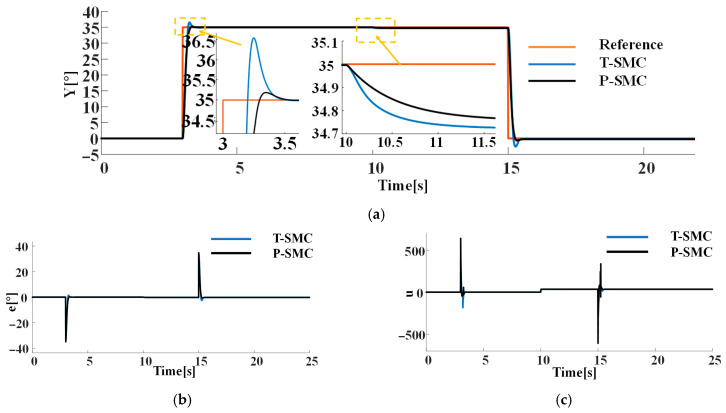
Comparison of P-SMC and T-SMC under the sudden disturbance $r_{d}\left(t\right)$: (**a**) the system output trajectories; (**b**) tracking error; (**c**) control input signals.

To validate the effectiveness of the proposed piecewise SMC method, it is compared against the T-SMC. The tracking performance of the two sliding mode control methods is shown in Figure 6.

As shown in Figure 6, following the introduction of the sudden disturbance $r_{d}\left(t\right)$ at 10 s, the proposed P-SMC confines the steady-state error within 0.3° and achieves 99.1% disturbance attenuation. Under the reference step change, T-SMC exhibits a peak overshoot of 4.31%. In contrast, the P-SMC employs a linear saturation within the boundary layer, reducing overshoot to 0.57%, thereby mitigating excessive inertial effects.

**Figure 7:**
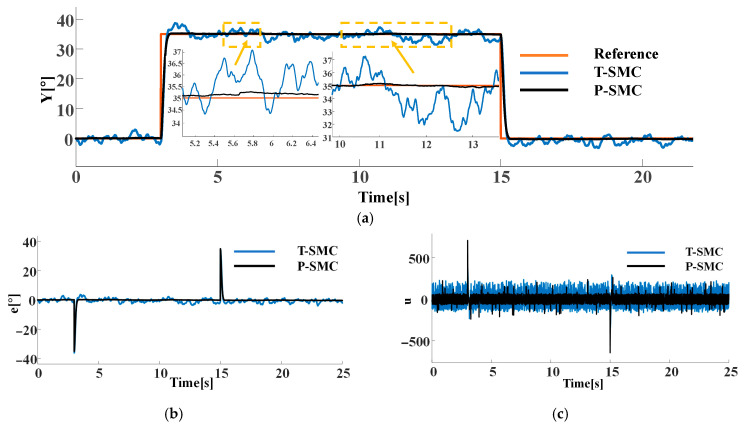
Comparison of P-SMC and T-SMC under the internal disturbance (Gaussian white noise) and measurement noise: (**a**) the system output trajectories; (**b**) tracking error; (**c**) control input signals.

In practical applications, both sensor measurement noise and internal system disturbances cannot be neglected, the former arises from limitations in measurement accuracy and high-frequency interference during signal transmission, while the latter may stem from structural tolerances, component wear and aging, and nonlinear friction effects. To more accurately simulate noise disturbances encountered in real operating conditions, Gaussian white noise with density $N_{0}=1$ is superimposed on both the system’s input and output channel in the simulation, thereby capturing the combined impact of internal and measurement noise. The response of each control strategy under this noise scenario is presented in Figure 7.

As illustrated in Figure 7, under high-noise conditions both sliding-mode controllers suffer from chattering, and high frequency control signal oscillations. However, the proposed P-SMC tightly maintains chattering within ±0.5° around the reference, significantly outperforming the T-SMC which chatters within ±5°. Notably, during the reference step transition, T-SMC not only produces large overshoot but also high-frequency chattering, with its control input spectrum concentrated in the high-frequency band under noise. In contrast, P-SMC employs boundary-layer saturation and a piecewise reaching law to effectively suppress chattering and limit overshoot. Moreover, the control signal of T-SMC presents severe high-frequency chattering, whereas P-SMC substantially reduces the oscillation amplitude by 58.1%.

**Figure 8:**
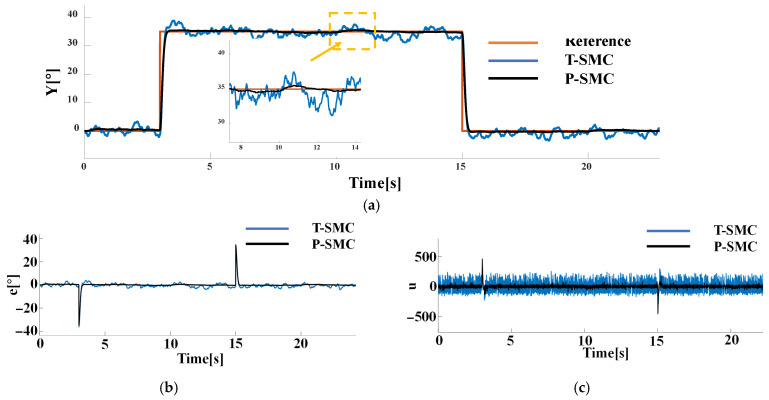
Comparison of P-SMC and T-SMC under the internal disturbance (periodic pulse) and measurement noise: (**a**) the system output trajectories; (**b**) tracking error; (**c**) control input signals.

**Figure 9:**
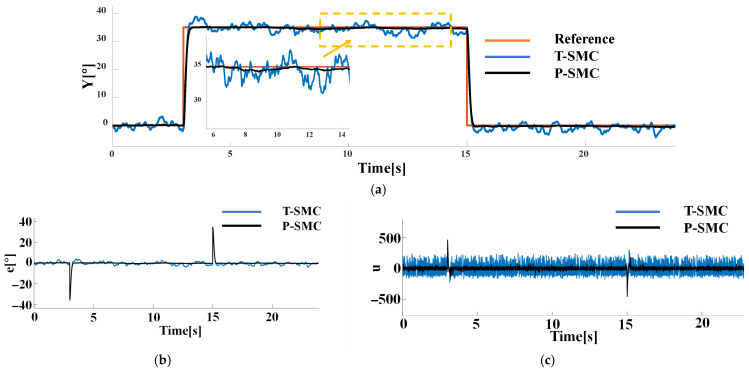
Comparison of P-SMC and T-SMC under the internal disturbance (pseudo random signal) and measurement noise: (**a**) the system output trajectories; (**b**) tracking error; (**c**) control input signals.

To further assess robustness under mixed-source disturbances, we fix measurement noise as Gaussian white noise with $N_{0}=1$ on the output channel and compare controller performance when the internal disturbance is (a) a periodic pulse with an amplitude of 10 and a period of 1 s or (b) a pseudo random signal with a mean of 0 and a variance of 20. The performance of each control strategy is shown in Figure 8 and Figure 9.

As illustrated in Figure 8 and Figure 9, both controllers successfully drive the system output to track the reference signal. The T-SMC exhibits significant chattering and steady-state oscillations, with oscillation amplitudes within ±5°. In contrast, the output of the P-SMC closely aligns with the reference signal, maintaining stable and accurate tracking with markedly reduced chattering. The results indicate that the P-SMC strategy effectively enhances system robustness against disturbances and unmodeled dynamics while achieving a higher precision of control.

These results clearly demonstrate the superior disturbance rejection and chattering suppression of the P-SMC, and the P-SMC controller delivers smoother control input signals and superior tracking accuracy under the dynamic disturbance. However, it is noticeably evident from the results that P-SMC exhibits significant high-frequency chattering in the control input signals under measurement noise, which is detrimental to actuator longevity and overall control loop stability.

### 5.3. Comparison of Improved ESO and Original ESO

**Figure 10:**
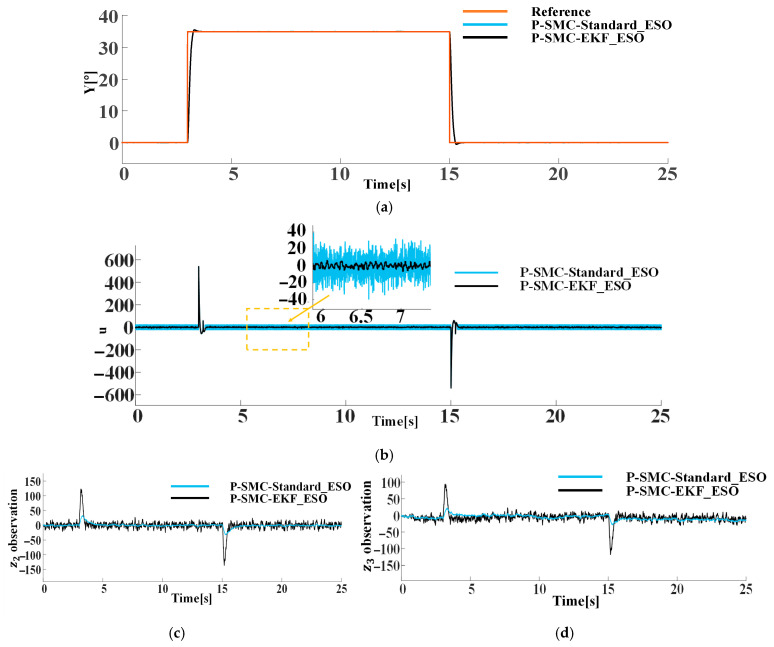
Comparison of EKF-ESO and standard ESO under the internal disturbance (Gaussian white noise) and measurement noise: (**a**) the system output trajectories; (**b**) control input signals; (**c**) observation of $z_{2}$; (**d**) observation of $z_{3}$.

Although the proposed P-SMC effectively suppresses high-frequency noise, its robustness under large disturbances remains limited. To address this, the sliding procedure is employed as an add-on to the baseline ADRC law, thereby enhancing overall disturbance rejection. Simultaneously, the Extend Kalman Filter (EKF) is integrated to refine the ESO’s output, increasing the sensitivity and accuracy of ESO in estimating system uncertainties. Under input and output channel contaminated with Gaussian white noise of density $N_{0}=0.1$, the control performance of ADRC augmented with sliding-mode compensation is compared using both the standard ESO and EKF-ESO schemes. Figure 10 illustrates the simulation results for both configurations under identical disturbance conditions.

The comparative simulation results indicate that, although the P-SMC-ADRC composite controllers based on the standard ESO and the EKF-ESO exhibit similar tracking speed and overshoot suppression, they differ markedly in control input signals smoothness and observer dynamic characteristics. With the standard ESO, the lagging estimation of the total disturbance causes the high-frequency oscillation. In contrast, the EKF-ESO is inherently more sensitive to rapid variations in system states and disturbances, as it amplifies the extended state estimates and responds swiftly to high frequency components due to the gain mechanism. These enhanced estimates are actively compensated by the NLSEF, resulting in 62.5% attenuation of oscillation amplitude and smoother actuator signals. Although the transient oscillations are induced in the observer states $z_{2}$ and $z_{3}$, the EKF-ESO rapidly converges and maintains significantly lower input signals oscillatory behavior. In summary, while the standard ESO delivers superior observer stability, the EKF-ESO based control method provides significantly smoother and more robust control action through the rapid, amplified estimation of EKF-ESO and compensation based on NLSEF, which trades transient observer oscillations for markedly smoother actuator inputs.

### 5.4. Comparison of the Overall Control Strategies

**Figure 11:**
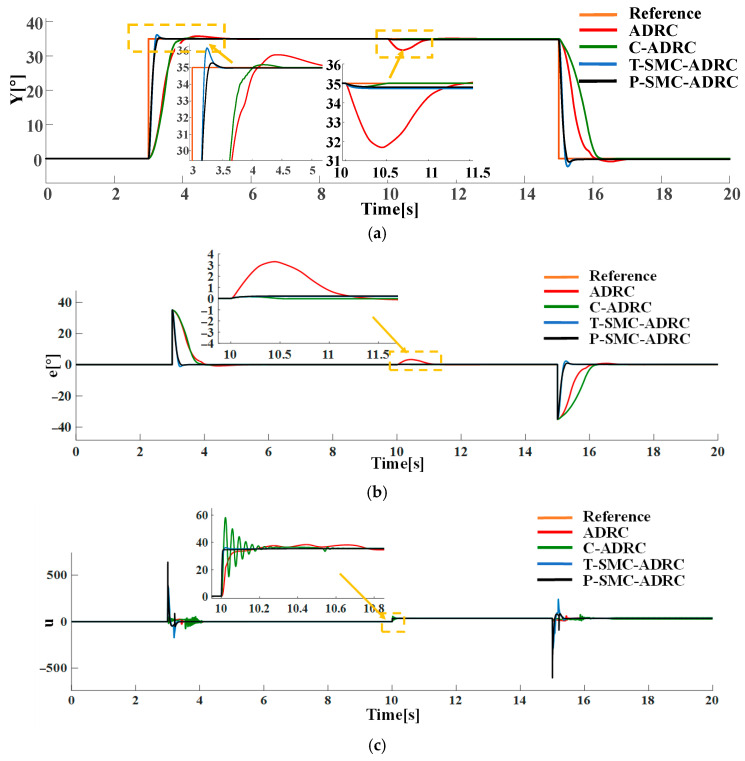
The comparative results under the step disturbance $r_{d}\left(t\right)$: (**a**) the system output trajectories; (**b**) tracking error; (**c**) control input signals.

To comprehensively assess the robustness of the proposed composite control strategy, four controllers were compared on the second-order nonlinear system (Equation (59)), the baseline ADRC, cascade ADRC (C-ADRC), composite control based on improved ADRC and T-SMC, and the proposed P-SMC-ADRC. Their tracking performance and control signals are systematically evaluated under these simulation scenarios:

Under the step disturbance $r_{d}\left(t\right)$, the tracking performance and control input signals of each strategy are compared in simulation. The results are shown in Figure 11.

**Figure 12:**
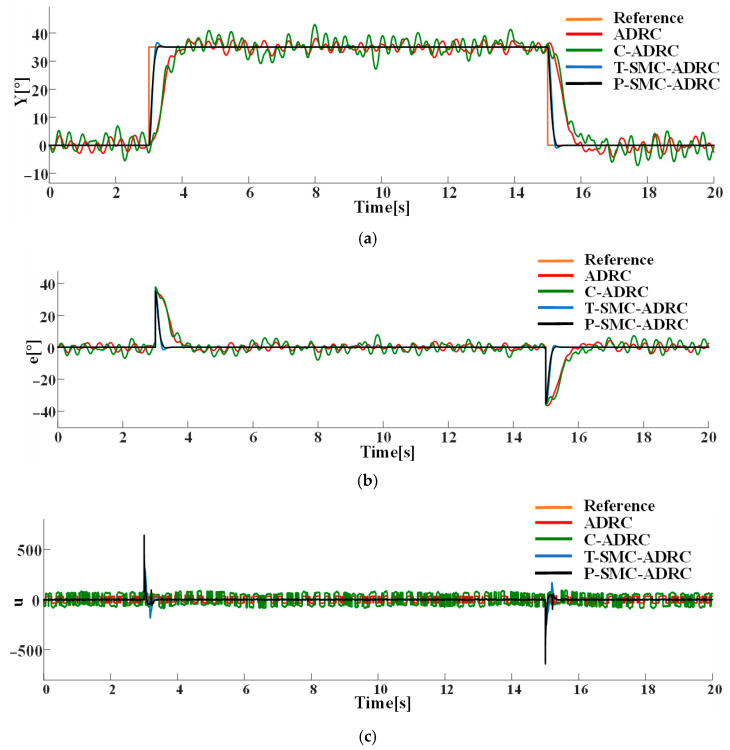
The comparative results under measurement noise of density $N_{0}=0.1$: (**a**) the system output trajectories; (**b**) tracking error; (**c**) control input signals.

With Gaussian white noise of density $N_{0}=0.1$ superimposed on the system output channel, the tracking performance and control signals of each strategy are compared in simulation. The results are presented in Figure 12.

**Figure 13:**
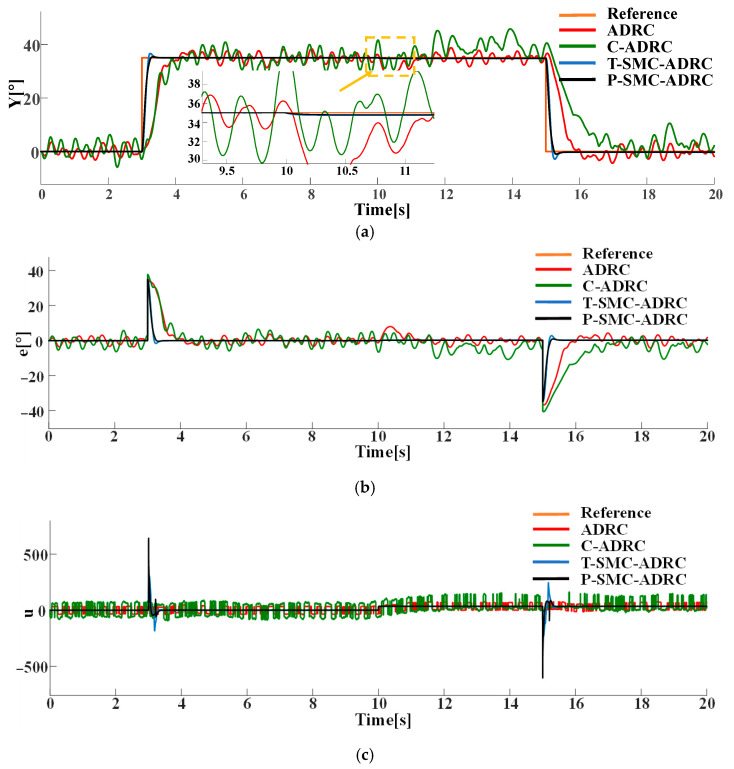
The comparative results under the input step disturbance $r_{d}\left(t\right)$ and measurement noise of density $N_{0}=0.1$: (**a**) the system output trajectories; (**b**) tracking error; (**c**) control input signals.

Under the combined conditions of the input step disturbance $r_{d}\left(t\right)$ and measurement noise of density $N_{0}=0.1$, the tracking performance and control input signals of each strategy are compared in simulation. The results are shown in Figure 13.

**Figure 14:**
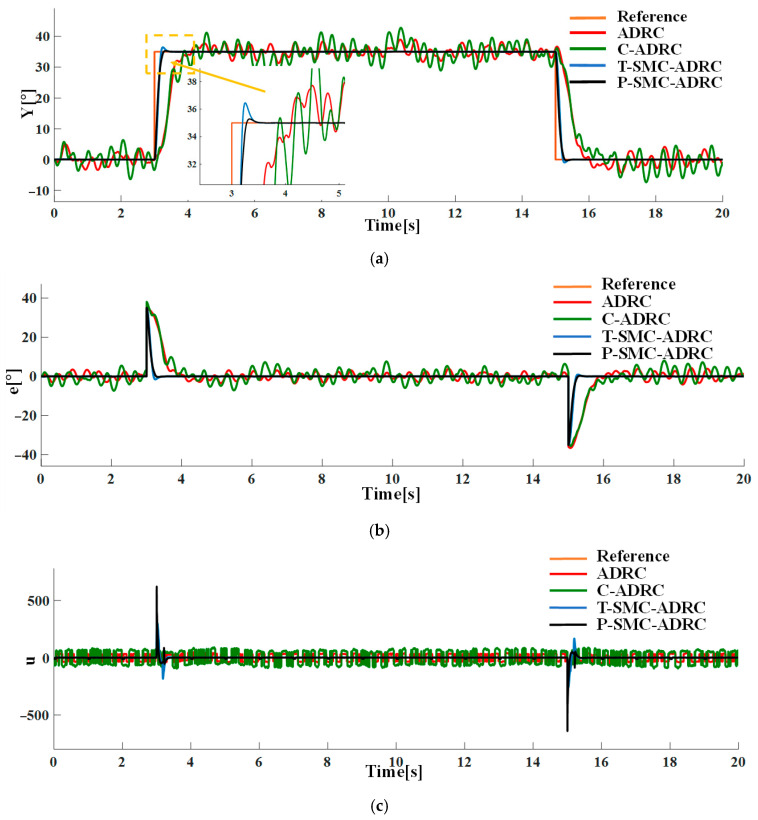
The comparative results under the input pulse disturbances and measurement noise of density $N_{0}=0.1$: (**a**) the system output trajectories; (**b**) tracking error; (**c**) control input signals.

Under the combined conditions of the input pulse disturbances and measurement noise of density $N_{0}=0.1$, the tracking performance and control input signals of each strategy are compared in simulation. The results are shown in Figure 14.

**Figure 15:**
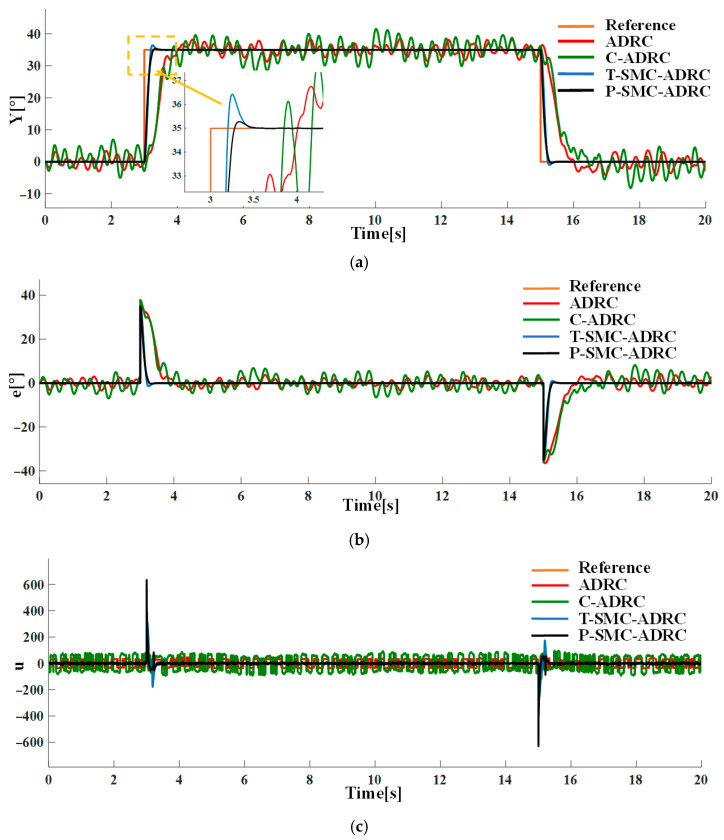
The comparative results under the input pseudo random signal and measurement noise of density $N_{0}=0.1$: (**a**) the system output trajectories; (**b**) tracking error; (**c**) control input signals.

Under the combined conditions of the input pseudo random signal and measurement noise of density $N_{0}=0.1$, the tracking performance and control input signals of each strategy are compared in simulation. The results are shown in Figure 15.

**Figure 16:**
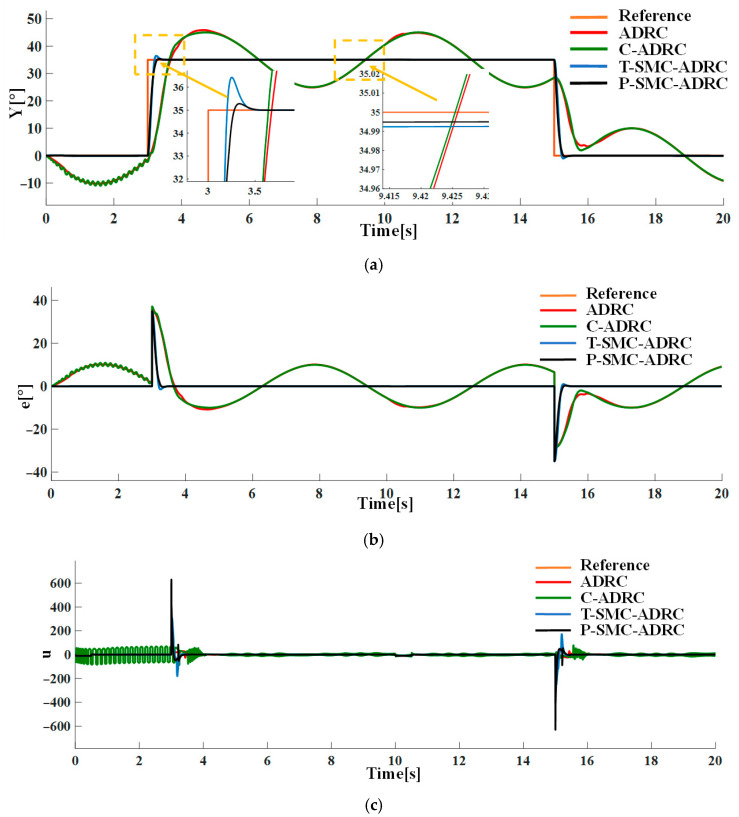
The comparative results under the input pulse disturbances and measurement noise of periodic Sinusoidal signal: (**a**) the system output trajectories; (**b**) tracking error; (**c**) control input signals.

Under the combined conditions of the input pulse disturbances and measurement noise of periodic Sinusoidal signal, the tracking performance and control input signals of each strategy are compared in simulation. The results are shown in Figure 16.

**Figure 17:**
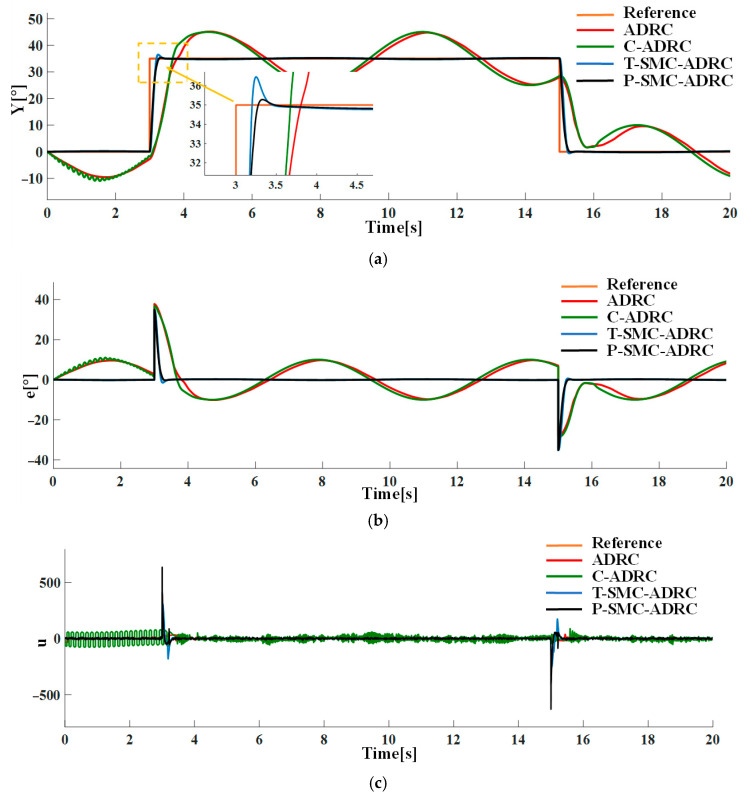
The comparative results under the input periodic Sinusoidal disturbances and measurement noise of periodic Sinusoidal signal: (**a**) the system output trajectories; (**b**) tracking error; (**c**) control input signals.

Under the combined conditions of the input periodic Sinusoidal disturbances and measurement noise of periodic Sinusoidal signal, the tracking performance and control input signals of each strategy are compared in simulation. The results are shown in Figure 17.

The simulation results demonstrate that the proposed P-SMC-ADRC delivers superior overall performance across all disturbance scenarios. The simulation results under the step disturbance $r_{d}\left(t\right)$ are shown in Figure 11. While C-ADRC achieves the fastest convergence in rejecting step disturbances, it incurs a prolonged settling time of 1.5 s and high-frequency oscillation in the control input signals. T-SMC-ADRC exhibits a substantial 4.54% overshoot. In contrast P-SMC-ADRC achieves convergence within 0.55 s with a maximum overshoot of only 0.91%. In the measurement noise scenario, which is illustrated in Figure 12, the standard ADRC and C-ADRC outputs persistently oscillate with sluggish response, and although T-SMC–ADRC shows some improvement, its overshoot remains significant. By contrast, P-SMC–ADRC markedly suppresses noise-induced oscillation, yielding the most stable output. The simulation results under combined step disturbance and measurement noise are shown in Figure 13. P-SMC–ADRC not only maintains rapid reference tracking with a maximum overshoot of only 0.97% but also achieves 99.4% disturbance attenuation and superior oscillation suppression, which effectively compensates for most measurement noise, outperforming all three benchmark controllers. As illustrated in Figure 14, Figure 15, Figure 16 and Figure 17, the proposed control methodology exhibits strong robustness and effective disturbance rejection under multiple types of disturbances from various sources. Additionally, in contrast to the control input signals shown in Figure 7c, the integration of the sliding mode control law with ADRC substantially mitigates input signals chattering, resulting in smoother control effort and superior overall performance.

Compared to the baseline ADRC, C-ADRC, and T-SMC-ADRC, the proposed P-SMC-ADRC achieves the smallest peak overshoot and converges more rapidly to the reference trajectory under step disturbances, while delivering the most effective oscillation suppression. Although a single control spike occurs at the instant of the step change, its amplitude quickly diminishes without inducing secondary overshoot, whereas the other methods exhibit sustained oscillations in steady state. These findings demonstrate that P-SMC–ADRC, by leveraging the high-gain rapid response of the piecewise sliding-mode law with boundary-layer saturation smoothing, together with precise total-disturbance compensation via ADRC and EKF-ESO, attains superior tracking accuracy and robustness in highly nonlinear systems subject to multi-source stochastic disturbances.

It is noteworthy that across all comparative experiments conducted in this study, the control input signals consistently exhibit transient high-gain-induced spikes during reference step changes. Although these spikes are of very short duration and do not cause secondary overshoot, they may still impose instantaneous stress on actuators. This phenomenon could raise reliability concerns for systems with stringent hardware longevity requirements, despite the strategy’s preservation of excellent dynamic performance and settling time. This is of particular concern in applications where actuator durability and long-term reliability are critical.

## 6. Conclusions

Addressing the challenge of insufficient response speed and robustness in optical attitude control systems subject to internal uncertainties and sensor measurement disturbances, a composite control strategy (P-SMC-ADRC) is proposed, which integrates the novel P-SMC with the improved ADRC based on EKF-ESO. The proposed P-SMC employs a power rate reaching law combined with linear feedback to rapidly drive the system state onto the sliding surface in the large error region, and switches to a linear sliding mode with a continuous saturation function to smooth the control input signals and suppress oscillation within the boundary layer. Moreover, to enhance the sensitivity to dynamic variations and disturbances, the EKF is incorporated into the ESO to amplify the response to system states and total disturbances. The enhanced estimates are actively compensated through Nonlinear State Error Feedback (NLSEF), resulting in smoother control input signals and superior disturbance rejection. Furthermore, PSO is employed to rapidly tune the key parameters of the composite control strategy, combining global search capability with fast convergence. Comprehensive simulations under step disturbances, measurement noise, and their combination demonstrate that the P-SMC–ADRC outperforms conventional ADRC, cascaded ADRC, and T-SMC–ADRC in key metrics that include a settling time of 0.55 s, a peak overshoot of 0.97%, approximately 99.4% disturbance rejection, and minimal control input signals oscillation, thereby confirming its superior disturbance rejection and robustness in highly nonlinear, multi-source stochastic environments.

## Figures and Tables

**Table 1:** The optimal parameters of the proposed P-SMC-ADRC.

$w_{0}$	$b_{0}$	α	γ	λ	μ	m	δ	ε
82.4737	11.3254	39.6540	29.7297	19.6342	0.25	31.2540	0.35	0.9

**Table 2:** The optimal parameters of the T-SMC.

σ	ρ	η	$b_{0}$
0.9	1.1354	0.8	16.4164

**Table 3:** The optimal parameters of the ADRC and C-ADRC.

$w_{0}$	$b_{0}$	$r_{0}$	$h_{0}$	*R*	*Q*
42.36	6.354	300	0.01	26.8133	Diag(1 ×10^−4^, 1, 1)

## Data Availability

Data are contained within this article.

## Abbreviations

The following abbreviations are used in this manuscript:

SMC	Sliding-mode control
ADRC	Active disturbance rejection control
TD	Tracking differentiator
NLSEF	Nonlinear state error feedback
ESO	Extended state observer
CESO	Cascade extended state observer
PSO	Particle swarm optimization
EKF	Extended Kalman filter
T-SMC	Nonsingular terminal sliding mode control
